# Theoretical Study on Fluorinated Derivatives of Sulfolane, Cyclopentanone, and Gamma-Butyrolactone

**DOI:** 10.3390/molecules28237770

**Published:** 2023-11-25

**Authors:** Sofja Tshepelevitsh, Agnes Kütt, Ivo Leito

**Affiliations:** Institute of Chemistry, University of Tartu, Ravila 14a, 50411 Tartu, Estonia; sofja.tsepelevits@ut.ee (S.T.); agnes.kutt@ut.ee (A.K.)

**Keywords:** fluorination, redox stability, basicity, electrochemistry, dielectric constant, isomers, COSMO-RS method, DFT calculations

## Abstract

In this paper, fluorinated compounds based on sulfolane, cyclopentanone, and gamma-butyrolactone are studied computationally, focusing on their applicability in electrochemical devices and acid–base-related studies. Candidates for solvents with (1) high polarity, (2) good electrochemical stability, and (3) low basicity were searched for. Some of the compounds are studied here for the first time. Electrochemical stabilities, dielectric constants, boiling points, basicities, and lipophilicities were estimated using DFT and COSMO-RS methods with empirical corrections. The effect of fluorination on these properties as well as the bond parameters was studied. The possible synthesis routes of the proposed compounds are outlined. Some molecules display a combination of estimated properties favorable for a solvent, although none of the studied compounds are expected to surpass acetonitrile and propylene carbonate by the width of the electrochemical stability window.

## 1. Introduction

Despite there being many solvents already in use for electrochemical applications [1,2], the search for new solvents and solvent additives is ongoing [3,4,5]. There are a number of properties that a compound must possess to be practically usable in electrochemical devices (batteries or supercapacitors) as a main solvent [2,5,6]:

▪ High redox stability (voltage durability), as the capacity of a double-layer capacitor is proportional to the square of the operating voltage.
▪ Low viscosity, which is a prerequisite for high ionic conductivity [7].
▪ High ion solubilities to produce electrolytes with sufficient ion concentrations (achievable by the high free energy of the solvation of relevant ions and high dielectric constant).
▪ Low enough melting point and high boiling and flash points.
▪ Sufficient thermal stability.
▪ Low protonation/deprotonation ability and low reactivity.

Additionally, low toxicity, low cost, and recyclability are desirable, if not strictly necessary. Compatibility with the electrode materials also plays a role, as some compounds that perform well with metal anodes may be incompatible with graphite anodes or vice versa.

Most properties that make an excellent electrolyte solvent are also needed in studies of strong acids and creating strongly acidic systems: high dielectric constant and solvation ability prevent undesirable interactions between ions, and a low reactivity allows for a wider choice of components. Low basicity is a necessary property for the study of strong acids, as the basic nature of the solvent has a leveling effect on the dissociation of acids and hinders differentiating their strengths. A sufficiently high boiling point is important for safety and retaining stable concentrations.

An ideal solvent hardly exists, as some of the listed properties are correlated in such a way that achieving two within the optimal range is difficult. The solvent’s ability for specific solvation, on the one hand, promotes salt solubility and ion separation; on the other hand, it usually comes with more pronounced acid–base properties and higher viscosity. For these reasons, protic solvents are generally unsuitable for the above-mentioned purposes. Most solvents currently used in nonaqueous electrochemistry are dipolar aprotic: carbonates, ethers, esters, and nitriles [1,2,8].

Fluorination can enhance some of the desirable characteristics of solvents, which, however, comes at the cost of some simultaneous undesirable changes. A variety of fluorinated compounds have been explored as solvents or electrolyte components for electrochemical devices, either experimentally [8,9,10,11,12,13,14] or theoretically [15,16,17]. Compared to nonfluorinated analogs, partially fluorinated compounds tend to have the following:

▪ Higher oxidation resistance due to a lower HOMO energy [9,18], but also a higher tendency to be reduced due to a lower LUMO energy [9,15].
▪ Lower flammability or even fire-suppressing properties [8,19,20].
▪ Higher dielectric constant, if the F atom is placed in the right position to increase the dipole moment of the molecule.
▪ Lower basicity and higher acidity.
▪ Higher viscosity due to an increase in molecular mass and hydrogen bond donicity [10,11,13], although, in some cases, viscosity reduction has been reported as well [21].

Most of the time, fluorinated (especially polyfluorinated) compounds are less reactive than their nonfluorinated analogs. However, in some cases, fluorination increases reactivity [15,16,22]. For example, alpha-fluorination weakens C-S bonds in sulfones [15,16], which contributes to the increase in reduction potential.

**Figure 1:**
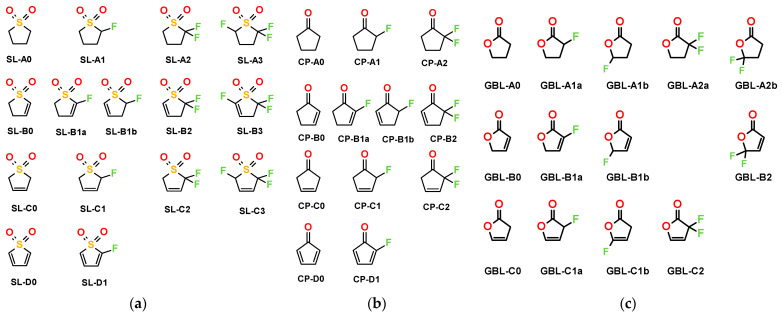
The studied molecules. The compound codes denote the parent structure (SL, GBL, or CP), number of double bond(s) (A—none, B/C—one, and D—two), and number of fluorine atoms. (**a**) Sulfolane derivatives, (**b**) cyclopentanone derivatives, and (**c**) GBL derivatives.

In this work, we choose three scaffold compounds—sulfolane (SL), cyclopentanone (CP), and gamma-butyrolactone (GBL)—to be modified by fluorination and the addition of double bonds and characterize the obtained molecules in the context of electrochemistry and studies of strong acids. The studied structures are shown in Figure 1.

Of the parent compounds, SL and GBL are used as electrolyte components [1,8]. SL has been suggested as facilitating the fast transport of lithium ions through the unique hopping diffusion mechanism [23].

The available literature data (Table 1) show that adding a double bond to a nonfluorinated parent structure dramatically increases the melting point (MP). Since the MP depends strongly on the compound purity, we abstained from making quantitative conclusions. However, it is clear that many compounds containing a double bond may not be usable as pure solvents but only in combination with lower-melting compounds. The effect of fluorination on the MP is not obvious since reduced symmetry and increased polarity have opposite influences on the MP. There are examples of both an increase and decrease in MP upon the addition of F atom(s) [13]. Considering the very low MP of the parent CP and GBL, it is likely that the solvents of the series CP-A and GBL-A are liquid at room temperature and usable in pure form.

Another potentially problematic aspect is the reactivity of the unsaturated compounds, which can limit their usability in harsh conditions and in combination with other reactive species. Most solvents that are typically used in electrochemical devices are composed of saturated molecules because double bonds compromise stability. However, unsaturated compounds can be used as electrolyte additives. They may be required to have a lower stability than the main solvent in order to react and form a film on the electrode surface [32,33,34].

The properties explored in this study are:

▪ Electrochemical stability windows (ESWs) were estimated via the calculated oxidation and reduction potentials in gas phase and DMSO, or approximated via the differences of the gas-phase HOMO and LUMO energies [35].
▪ Dielectric constants (*ε*_r_) and boiling points (BP) were computed with the COSMO-RS method with empirical corrections.
▪ Basicities were characterized via the predicted Gibbs energies of the transfer of a proton from water to the studied solvent (∆_tr_*G*°(H^+^)), basicity in acetonitrile (p*K*_aH_(MeCN), corresponding to the p*K*_a_ of the protonated molecule), and gas-phase basicity (GB) of the solvents.
▪ Mutual solubility with water and lipophilicity were computed with the COSMO-RS method.

*ε*_r_ values, ∆_tr_*G*°(H^+^) values, and solubilities were computed on the assumption that the compounds are liquid at room temperature (which, as explained above, this may not always be the case).

## 2. Results

The results of the computations, together with some values from the literature, are presented in Table 2.

## 3. Discussion

### 3.1. Effect of Fluorination on Bond Strengths

**Figure 2:**
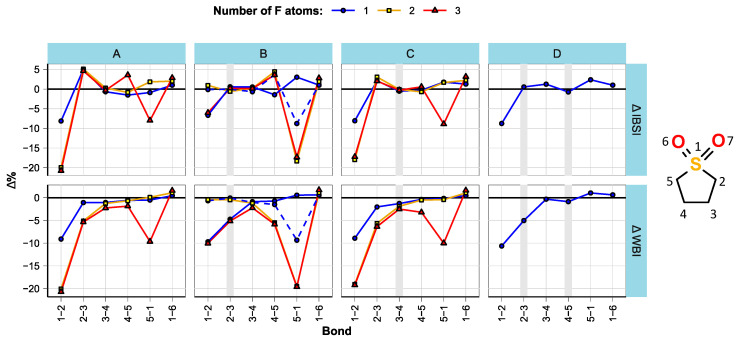
Effect of fluorination on bond strength and multiplicity in SL derivatives. The plot is split by structural subgroups (**A**–**D**), and double bonds are marked with wide vertical lines. The compound SL-B1b (mono-fluorinated at C5) is marked by a dashed line. The parameters of the S=O bonds 1-6 and 1-7 were averaged.

**Figure 3:**
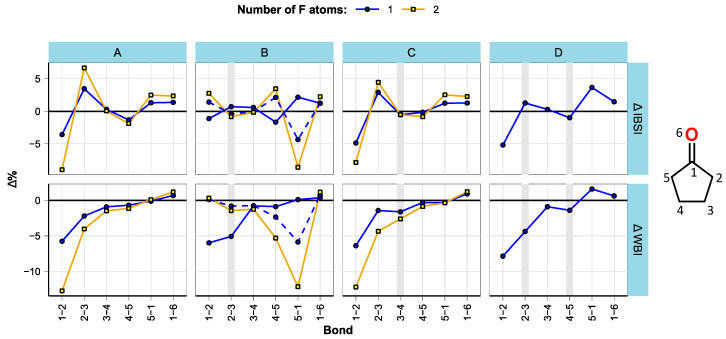
Effect of fluorination on bond strength and multiplicity in CP derivatives. The plot is split by structural subgroups (**A**–**D**), and double bonds are marked with wide vertical lines. The compound CP-B1b (mono-fluorinated at C5) is marked by a dashed line.

**Figure 4:**
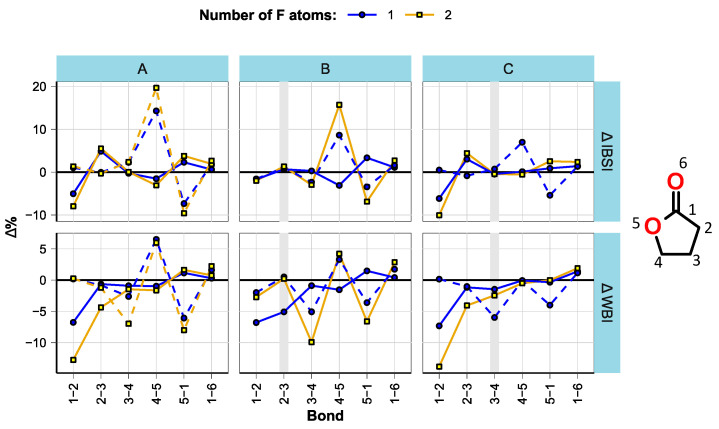
Effect of fluorination on bond strength and multiplicity in GBL derivatives. The plot is split by structural subgroups (**A**–**C**), and double bonds are marked with wide vertical lines. Solid lines denote the compounds fluorinated at C2 and dashed lines those at C4.

The bond parameters were analyzed in a similar manner to ref. [16]. As expected, bond lengths were found to be well correlated with the bond strengths within compound groups. The estimated changes in bond strengths (expressed as intrinsic bond strength indices (IBSI) [41]) and multiplicities (expressed as Wiberg bond indices (WBI) [42]) with fluorination are shown in Figure 2, Figure 3 and Figure 4. All bond length, WBI, and IBSI values are provided in Table S5.

It was found that, in all studied compound groups, α-fluorination weakens the bond between the fluorinated carbon and electronegative group and reduces its bond order. At the same time, it can strengthen other bonds. Also, a slight strengthening of the C=O and S=O bonds was observed. In sulfolanes (Figure 2), the bond weakening upon fluorination is the most pronounced, and the effect of the second F atom is similar to the effect of the first. This is in agreement with the literature [16]. The strengthening of the C2-C3 bond, especially noticeable when the bond is single, is indicated by the increased IBSI; however, this is at odds with the reduced WBI of the bond.

Similar effects were observed for cyclopentanone derivatives (Figure 3). α-fluorination weakens the C1-C2 bond, while slightly strengthening the C1-C5 bond and possibly the C2-C3 bond. Fluorination at C5 weakens the C1-C5 bond, while slightly strengthening the C1-C2 and C4-C5 bonds. The effect of the second F atom on bond weakening is mostly similar to that of the first F atom, but its effect on bond strengthening tends to be weaker.

In GBL derivatives (Figure 4), α-fluorination weakens the bond between C2 and C1 (carbonyl), while fluorination at C4 strengthens the C4-O5 bond but weakens the C1-O5 bond. The increase in the IBSI of C4-O5 is outstanding compared to the SL and CP derivatives, being over 10% for subgroup A (no double bonds). The effect of the second F atom is mostly weaker than that of the first. 

### 3.2. Effect of Structural Variations on the Electrochemical Stability

**Figure 5:**
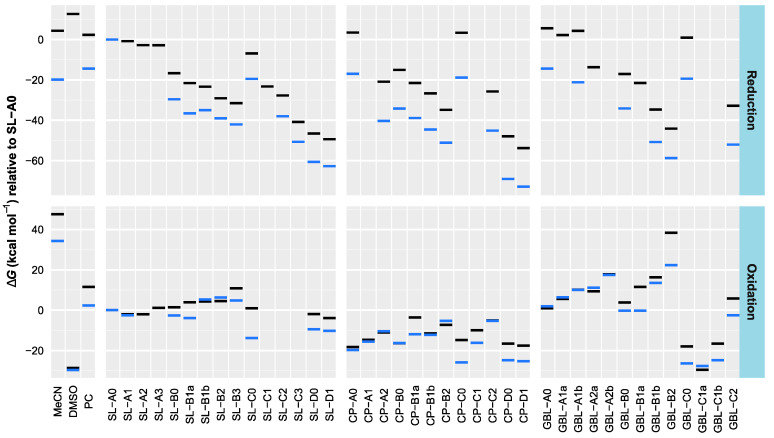
Computed oxidation and reduction free energies in the gas phase and DMSO relative to sulfolane (**SL-A0**). Black lines—gas phase; blue lines—DMSO. The values are not shown if, in any of the involved calculations, the structure broke apart or was rearranged during geometry optimization.

**Figure 6:**
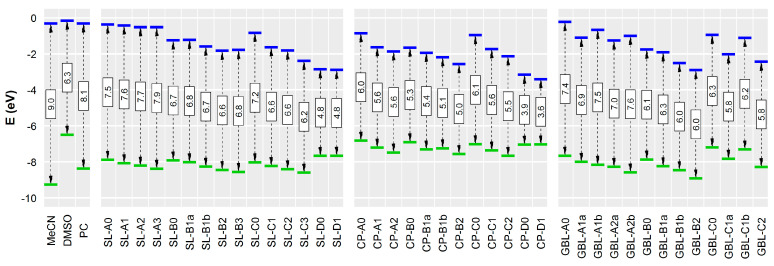
HOMO and LUMO energies computed at the B3LYP/6-311+G** level of theory. Blue lines—LUMO levels; green lines—HOMO levels.

The calculated oxidation and reduction free energies in the gas phase and DMSO (as an example of a polar aprotic environment) are presented in Figure 5. The values are presented relative to those of sulfolane (**SL-A0**). For several compounds, the geometries of the oxidized/reduced species were broken up or rearranged during the geometry optimization step of the computation, hinting at the low stability of these species (such geometries were not used for further computations). It can be seen that, first, solvation in DMSO does not significantly change the stability trends compared to the gas phase (Figure 5), and second, HOMO and LUMO levels obtained with the B3LYP functional adequately describe the trends in stability (Figure 6).

The computations show that fluorination (both number and position of F atoms) has a relatively small effect on the compound’s propensity to oxidation or reduction, compared to the significant drops in reduction stability that accompany the additions of the double bond(s) adjacent to the polar group (series B and D, and also C for GBL derivatives). The structural variations in the studied molecules influence reduction more than oxidation. Most studied molecules have lower reduction stabilities than MeCN, PC, and DMSO. None of the compounds have resistance to oxidation that is superior to MeCN, although in this respect, many outperform DMSO, and several GBL group compounds outperform PC. 

As the addition of fluorine tends to lower both HOMO and LUMO levels in the studied compounds, the net effect of fluorination can be both positive and negative, depending on the structure. In most studied cases, however, fluorination slightly narrowed ESW.

### 3.3. Effect of Structural Variations on Other Properties

**Figure 7:**
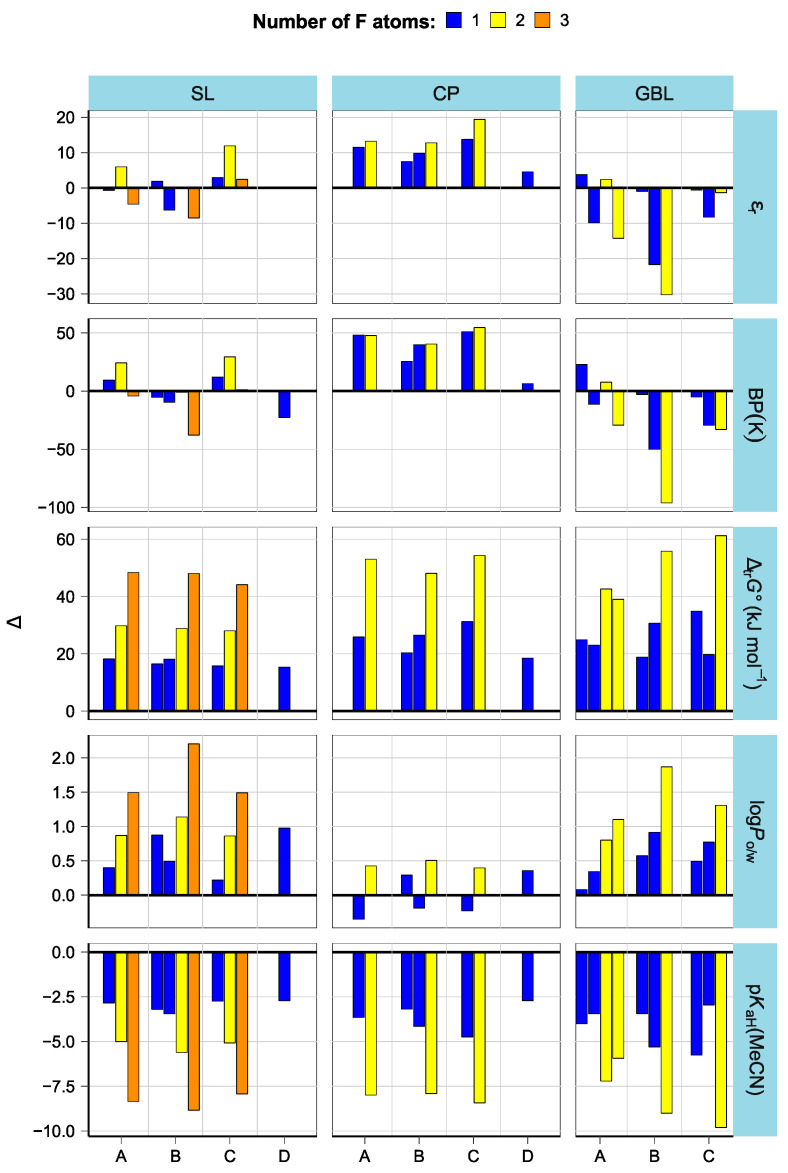
Effect of fluorination on compound properties. On the *x*-axis are the structural subgroups (A—no double bonds, B/C—one double bond, and D—two double bonds).

As seen in Figure 7 and Table 2, the effect of fluorination on compound properties varies according to compound group. The dielectric constant increases in the CP group but decreases in the GBL group. The likely reason is the different effects of fluorination on the dipole moment of the molecules. In the studied CP derivatives, fluorination always increases the dipole moment, while in GBL, the dipole moment can change both ways depending on substitution. As in the case of bond strengths, the effect of F atoms is not additive. The changes in *ε*_r_ values in the SL group are within the prediction uncertainty. A similar situation is observed with the boiling points of fluorinated compounds: an increase in CP, a decrease in GBL, and small and inconsistent changes in SL. ∆_tr_*G***°**(H^+^) values are invariably increased by fluorination, and the effect of F atoms appears to be more or less additive. Lipophilicities (expressed as octanol/water partition coefficients) are notably higher for fluorinated GBL and SL derivatives, but the effect is weaker and less consistent in the CP group. A more pronounced increase in the dipole moment of CP likely counters the lipophilicity-inducing effect of the F atoms. The patterns of p*K*_aH_(MeCN) match the inverted patterns of ∆_tr_*G***°**(H^+^). Each F atom decreases the basicity by 2.5–3 orders of magnitude.

The differences between the positional isomers of the fluorinated compounds were rather insignificant in SL and CP groups and only became notable in GBL-type scaffolds with double bonds (**GBL-B1a** vs. **GBL-B1b**, and **GBL-C1a** vs. **GBL-C1b**). While the differences in *ε*_r_, BP, and log*P*_o/w_ matched the differences in molecular dipoles (**GBL-B1b** and **GBL-C1b** have lower dipole moments), the differences in basicity can be attributed to differences in electron delocalization: **GBL-B1b** and **GBL-C1a**, where F is located at the sp3 carbon, are less basic than their isomers, where F is conjugated with O atoms.

### 3.4. Solubility Trends

**Figure 8:**
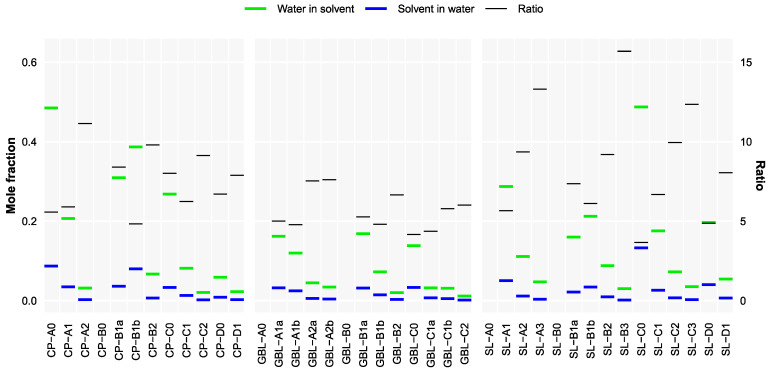
Calculated solubility estimates (in mole fraction) and their ratios on the assumption that the solvent is liquid. No values are provided if miscibility was predicted.

Figure 8 demonstrates the predicted solubility values of the studied compounds in water and of water in the studied compounds. It must be stressed that, if the compound is solid at room temperature, then the absolute values may be significantly overestimated, as the computation does not take into account the Gibbs energy of fusion. However, these predictions successfully demonstrate the hydrophobic effect of the added fluorine atoms. The solubilities of the studied compounds in water are more affected by fluorination than the solubility of water in them. While fluorination may increase polarity, which in itself facilitates solubility in polar media, this effect is clearly weaker than that of the larger size and low polarizability of fluorine atoms.

### 3.5. Synthesis of the Proposed Compounds

Sulfolane (**SL-A0**), 1,1-dioxide-2,3-dihydro-thiophene (**SL-B0**), 3-sulfolene (**SL-C0**), and 1,1-dioxide-thiophene (**SL-D0**) are widely available compounds whose synthesis originates either from alkenes (with the insertion of SO_2_) [43], from thiophenes (generally using H_2_O_2_ as an oxidizer) [44,45,46], or by catalytic double bond migration procedures (with transition metal catalysts) [47,48]. Their fluorinated derivatives can therefore be synthesized in a similar way using fluorinated alkenes (**SL-A2**, **SL-C2**), thiophenes (**SL-D1**) [49], or fluorinated 1,1-dioxide-2,3-dihydro-thiophenes (**SL-B1**, **SL-B2**) as precursors. Additionally, fluorination using a gaseous F_2_/N_2_ mixture of parent compounds has been employed (**SL-A1**) [50]. Perhaps **SL-C1** could be prepared similarly. The preparation of the tri-fluorinated analogs **SL-A3**, **SL-B3**, and **SL-C3** could be challenging by the abovementioned methods because the respective fluorinated alkene and thiophene are not available and their preparation methods have not been published.

γ-Butyrolactone (**GBL-A0**) can be fluorinated using F_2_/N_2_ gas [51] to obtain a mixture of **GBL-A1a** and **GBL-A1b**. The deoxyfluorination of hydroxy-γ-butyrolactone is also a well-established procedure [52]. The preparation of **GBL-A2** was not described, but the preparation could be considered from 2,2-difluorobutanedioic acid [53,54], 2,2-difluoro-1,4-butanediol by cyclization [55], or from 3,3-difluorodihydro-2,5-furandione [55] by hydrogenation. These precursors are readily available compounds. The fluorination of parent compounds similar to ethylene carbonate may be possible [56]. **GBL-B1a** [57] and **GBL-B1b** [58] are readily available compounds that can be prepared via cyclization. The preparation of **GBL-B2** by the isomerization of fumaroyl fluoride seems to be rather complicated [59]. Methods for fluorinated 2(3H)-furanone (**GBL-C0**) derivatives have not been published; however, synthesis from fluorinated precursors or double bond migration could be employed [60].

Cyclopentanone (**CP-A0**) is fluorinated to obtain a mono-fluorinated derivative (**CP-A1**) using different fluorinating agents (Accufluor, XeF_2_, etc.) [61,62,63]. **CP-A2** is a readily available (although expensive) compound. Its synthesis methods have not been published, but synthesis from difluoro adipic acid esters may be possible [64]. Cyclopentenone **CP-B1a** is prepared using F_2_/N_2_ gas [65]. Methods for other cyclopentenone derivatives, as well as methods for fluorinated cyclopentadienone (CP-D1), are absent in the literature. Using fluorinated precursors or different fluorination methods should be applicable to obtain some degree of fluorination.

## 4. Conclusions

**Figure 9:**
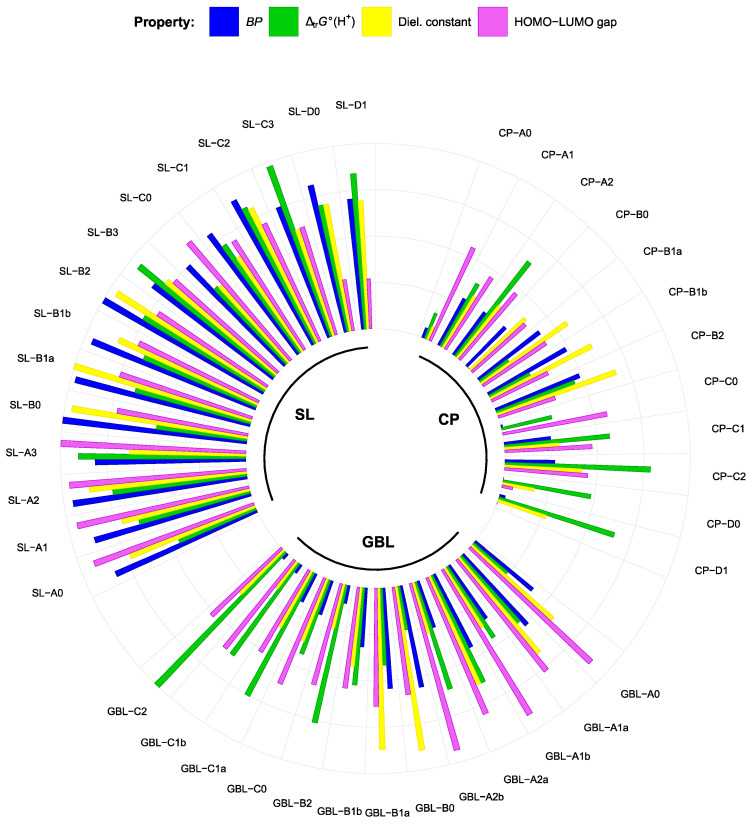
Normalized values of the essential properties of the studied compounds (for each property, the values of all represented compounds were rescaled to the range from 0 to 1).

The problem when choosing suitable solvents for nonaqueous applications involving electrochemistry and/or strong acidity is that many beneficial solvent properties are correlated with unfavorable ones. Therefore, trade-offs have to be found. Figure 9 illustrates the relative advantages of the studied compound groups in terms of the parameters that are essential for the main solvent: BP (well correlated with flash points [66]), ∆_tr_*G***°**(H^+^) values as estimates of solution basicity (higher values indicate lower basicities), and dielectric constant and HOMO-LUMO gap as a first estimate of electrochemical stability. As expected, sulfolanes (especially subgroups A and B) surpass the other groups in terms of a favorable combination of properties: ESWs are estimated to be wide, BP and *ε*_r_ are relatively high, and basicities are medium to low. CP are the least suitable main solvent candidates given their relatively high basicities, low boiling points, and *ε*_r_ and HOMO-LUMO gaps. Some of GBL derivatives (series A and B) look promising with wide ESW, high *ε*_r_, and medium basicity.

The presence of double bonds (subgroups B–D) is expected to significantly increase melting points and decrease reduction stability, making the respective compounds less likely to be suited for the role of main solvent and more eligible for the role of reactive electrolyte additive.

## 5. Materials and Methods

### 5.1. Computational Parameters and Software

Gas-phase properties were computed with the Gaussian 16 Rev. A.03 software [67]. The geometries of all the conformers of uncharged species were created and optimized using DFT at the M06-2X/6-311+G** and B3LYP/6-311+G** levels of theory. The geometries of reduced/oxidized species were optimized at the M06-2X/6-311+G** level of theory. For the calculation of solvation free energies in DMSO, geometry optimization for uncharged and reduced/oxidized species was carried out at the M06-2X/6-31+G* level in the gas phase and using the SMD model [68] (solvent DMSO). For all optimized geometries, vibrational spectra were computed to ensure that the optimized geometries correspond to the true energy minima. Small imaginary frequencies in two calculations (compounds **SL-C2** and **CP-B2**) could not be removed by reoptimization and were ignored. 

Oxidation and reduction free energies (∆_ox_*G* and ∆_red_*G*) for molecule S in the gas phase were computed from the lowest-energy conformers at the M06-2X/6-311+G** level of theory, as follows:

∆_ox_*G*(S^0^) = *G*(S^+^)_gas_ − *G*(S^0^)_gas_ (1)

∆_red_*G*(S^0^) = *G*(S^–^)_gas_ − *G*(S^0^)_gas_ (2)

Solvation free energies (∆_solv_*G*) were calculated as the differences of the free energies of the molecules in DMSO and in the gas phase, computed at M06-2X/6-31+G* (with and without the SMD model, respectively).

Oxidation and reduction free energies in DMSO were calculated as follows:

∆_ox_*G*(S^0^)_DMSO_ = *G*(S^+^)_gas_ + ∆_solv_*G*(S^+^) − *G*(S^0^)_gas_ − ∆_solv_*G*(S^0^) (3)

∆_red_*G*(S^0^)_DMSO_ = *G*(S^–^)_gas_ + ∆_solv_*G*(S^–^) − *G*(S^0^)_gas_ − ∆_solv_*G*(S^0^) (4)

The E_HOMO_ and E_LUMO_ values were obtained from the lowest energy conformers at the B3LYP/6-311+G** level of theory. 

Wiberg bond indices (WBI) [42] and intrinsic bond strength indices (IBSI) [41] were computed with the Multiwfn software (version 3.8, in development, accessed on 9 June 2023) [69] using the results calculated by the B3LYP/6-311+G** method.

GB values were computed with the G4MP2 method [70].

The COSMO-RS method was used for computing *ε*_r_, p*K*_aH_, Δ_tr_*G***°**(H^+^), BP, log*P*_o/w_ values, and solubilities. The geometries of all conformers and protomers of the studied solvents were optimized at the BP86/TZVP level of theory in an ideal conductor (COSMO model), followed by single-point energy calculation at the BP86/def2-TZVPD level of theory with the Fine cavity parameter. Vibrational spectra were computed to ensure that the obtained geometries correspond to energy minima. Solvent properties were computed from the obtained surface charge distributions using the COSMOtherm software (release 2023) [71] using all conformers of the involved species. DFT computations were carried out using Turbomole V6.5 software [72].

### 5.2. Solvation Models

COSMO-RS (Conductor-Like Solvation Model for Real Solutions) [73,74,75,76] is a method based on DFT computations and statistical thermodynamics that can be used for modeling arbitrary multicomponent fluids. As a first step, the geometries of all involved species are optimized in the ideal conductor, which yields the partial charge distribution on the molecular surface and energy of the structure. In the second step, the interactions of the molecules in the fluid mixture are accounted for via the pair-wise interactions of their surface segments. This allows access to a variety of properties determined by the free energies of molecules in the studied media (liquid–vapor diagrams, partition coefficients, solvation energies, etc.). Unlike most other solvation models, COSMO-RS is parametrized at the atomic level and is suitable for modeling novel substances for which no experimental data exist to date.

SMD is an implicit solvation model based on solute electron density [68]. The solvent is represented as a dielectric continuum at the solute–solvent boundary. SMD provides solvation energies comparable in quality to those obtained with COSMO-RS, but is not usable for arbitrary liquids as its parametrization relies on the experimental data of the solvent.

### 5.3. Accuracy of the Computed Values and Applied Corrections

**Figure 10:**
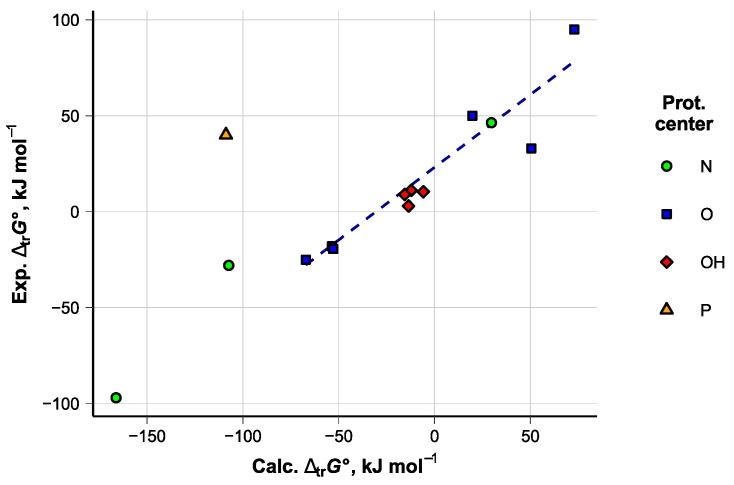
Relationship between the experimental and computed ∆_tr_*G*(H^+^) values. The dashed line is the regression line for the aprotic solvents protonating at the O atom. The numerical data are provided in Table S1.

∆_tr_*G*°(H^+^): This property is used as the basicity estimate of the solvents and is defined as the Gibbs energy change i the following process (Equation (5)):

(H_3_O^+^)_H2O_ + (S)_S_ ⇄ (H_2_O)_H2O_ + (SH^+^)_S_ (5)

where S denotes the studied solvent and the subscripts denote the corresponding environment. The ∆_tr_*G*°(H^+^) values of the process for several solvents were computed with the COSMO-RS method and correlated with the values from ref. [38] (details in Table S1). The results are shown in Figure 10. While the absolute errors of the computed values are significant, the experimental and computed values correlate satisfactorily within the compound groups with the same protonation center. The correction equation for the solvents of interest (Equation (6)) was composed using the six aprotic solvents protonating on the O atom. Standard deviations of the regression parameters are provided in parentheses. *S*_res_ refers to the overall regression standard deviation (standard deviation of the residuals). Given the large value of *S*_res_, the predicted ∆_tr_*G***°**(H^+^) values can be considered only semiquantitative. The liquid state of the solvent was assumed in all calculations.

∆_tr_*G***°**_Exp_(H^+^) = ∆_tr_*G***°**_Calc_(H^+^) · 0.76(0.13) + 23.1(7.2)
*N* = 6, *R*^2^ = 0.89, *S*_res_ = 18 kJ mol^−1^ (6)

**Figure 11:**
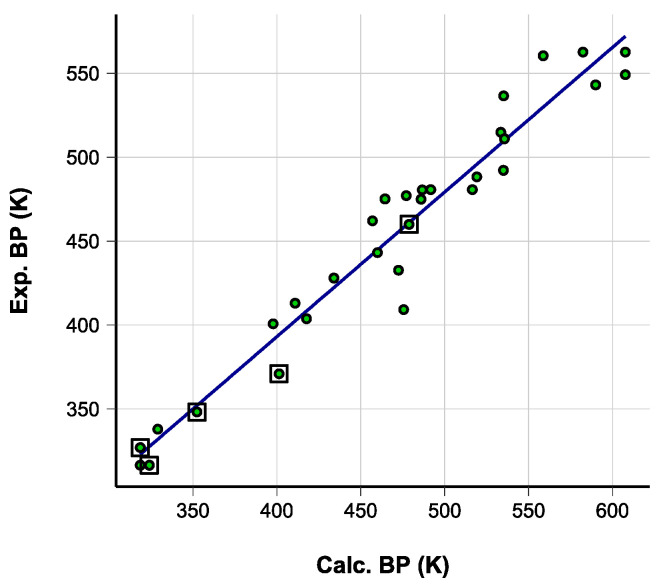
Correlation between the experimental and computational BP values. Fluorinated solvents are marked with squares.

BP: The accuracy of the boiling point values computed with COSMO-RS was assessed using a set of solvents containing structural features similar to the studied compounds and covering the expected span of BP values (Figure 11, Table S2). Equation (7) was used to correct the values systematically overestimated by computations. The standard errors of the regression parameters are provided in parentheses. The estimated standard uncertainty of the corrected values was 18 K. The data show that the effect of fluorination is predicted acceptably well, considering the overall accuracy. The magnitude of the effect of adding double bonds, however, tends to be overestimated.

*BP*_Exp_ = *BP*_Calc_ · 0.86(0.04) + 48(21)
*N* = 29, *R*^2^ = 0.94, *S*_res_ = 18 K (7)

Free Energies of Oxidation and Reduction: The functional M06-2X has one of the best performances to predict the gas-phase ionization potentials and electron affinities amongst the popular DFT functionals, with mean unsigned errors as low as 10 and 13 kJ mol^−1^, respectively [77]. For the SMD model, the mean unsigned errors of the solvation free energies in DMSO computed at the M05-2X/6-31G* level of theory were estimated as 0.64 kcal mol^−1^ for neutrals and 4.5 kcal mol^−1^ for anions [68]. With the level of theory used in this work, we would expect the errors to be even lower. SMD was shown to perform decently in the prediction of aqueous reduction potentials, outperforming COSMO-RS for aprotic solutes [77].

Using HOMO and LUMO levels in this work relied on the following approximations [35,78]:

∆_ox_*G* ≈ *IP* ≈ −*E*_HOMO_ (8)

∆_red_*G* ≈ *EA* ≈ −*E*_LUMO_ (9)

where ∆_ox_*G* and ∆_red_*G*—free energy of oxidation/reduction, *IP*—ionization potential, and *EA*—electron affinity. *E*_HOMO_ and *E*_LUMO_ depend considerably on the computational method but correlate well with the oxidation and reduction potentials, respectively [3]. 

Dielectric constant (*ε*_r_): The accuracy of dielectric constant estimates by the COSMO-RS method was assessed using the set of 20 solvents presented in Table S3. The solvents were selected by similarity to the compounds of interest: each contains one ring, no N atoms, and preferably one or more F atoms. The correction Equation (10) was obtained (standard deviation of regression parameters are shown in parentheses):

log_10_(*ε*_r_, exp.) = log_10_(*ε*_r_, calc.) · 1.12(0.05) + 0.01(0.05)
*N* = 20, *R*^2^ = 0.97, *S*_res_ = 0.08 log units (10)

To verify the reliability of the approach, *ε*_r_ values of some structurally assorted solvents were predicted (Table S3, Figure S1). The results demonstrate that the predictions are mostly accurate at moderate *ε*_r_ ranges, but *ε*_r_ values of around or over 40 can be considerable in error and more likely under- than overestimated. The *ε*_r_ values for fluoropyridines and dimethylsulfate were strongly underestimated. For solvents structurally similar to the model compounds, we considered predictions as quantitative at *ε*_r_ < 35 (RMSE ca. 4) and semiquantitative at *ε*_r_ > 35 (RMSE > 10). The semiquantitative nature of the prediction at high *ε*_r_ values is not a problem because *ε*_r_ values above 35 are certainly suitable for the purposes of this study; thus, knowing the accurate *ε*_r_ value is not critically important.

p*K*_aH_: The p*K*_a_ and p*K*_aH_ values predicted by COSMO-RS are usually biased, yet well correlated with the respective experimental data. With a lack of suitable experimental data to evaluate or correct the basicity values in acetonitrile (p*K*_aH_(MeCN)), we suggest that these values be used for semiquantitative comparison between structurally similar compounds rather than to access absolute basicities.

log*P*_o/w_: COSMO-RS is known to predict the octanol–water partition coefficients very well, with some rare exceptions [79,80]. The standard error of the predictions is conservatively estimated as ca. 0.3 units; therefore, no corrections are needed. The values correspond to wet octanol at 25 °C.

Mutual Solubility with Water: The reported accuracy estimates for the aqueous solubility predictions by COSMO-RS vary by solute type and the parametrization used. The expected error for liquid solutes is 0.3 [81] log units or lower (0.1 log units in ref. [82]). However, for solid solutes, it is 0.5 [81] log units or higher [82]. Dupeux et al. [80] reported an RMSE of 0.18 log units for a set containing liquid and solid solutes; the higher accuracy may be partly due to the improved method parametrization. COSMO-RS predicted the solubility trends of water in various hydrocarbons quite well; the relative bias of the predicted mole fractions was within 29% [83]. For many studied compounds, we do not know with certainty whether they are liquid or solid at room temperature. The solubility estimates assume a liquid state of the solvents. The results were obtained using the “Liquid Extraction” option of COSMOtherm at 25 °C. No corrections were used.

Note on Viscosity Computations: COSMO-RS predicted the viscosities of a set of typical electrolyte solvents with a mean absolute deviation of 0.22 cP and a good correlation between the predictions and experimental data [35]. However, a test using the available data on fluorinated solvents and their nonfluorinated analogs showed that the effect of fluorination is not well reproduced by computations. It is possible that the random errors are of similar magnitude to the real effect of fluorination. Thus, viscosities were not reported in this work.

## Figures and Tables

**Table 1:** Literature data on the melting points (°C) of some of the studied compounds.

Double Bonds	Compound	MP	Compound	MP	Compound	MP
0	**SL-A0**	28.4 [24]	**CP-A0**	−52.8 [25]	**GBL-A0**	−43.1 [26]
1	**SL-B0**	49–50 [27]			**GBL-B0**	4–5 [28]
1	**SL-C0**	63–64 [29]			**GBL-C0**	48–49 [30]
2			**CP-D0**	96–98 [31]		

**Table 2:** Estimated properties of the investigated molecules. ∆_ox_*G* and ∆_red_*G* values are relative to sulfolane (**SL-A0**). Where available, experimental values are provided with reference to the source. Three conventional solvents were added for comparison (MeCN—acetonitrile, DMSO—dimethyl sulfoxide, PC—propylene carbonate). Further computed data (p*K*_aH_(MeCN), GB, and solubility estimates) are available in Table S4.

Compound	CAS	Relative Red./Ox. Energies (kcal mol^−1^)				E_HOMO_ (eV)	E_LUMO_ (eV)	*ε*_r_	*BP* (K)	∆_tr_*G*°(H^+^) (kJ mol^−1^)	log*P*_o/w_
		∆_ox_*G*_gas_	∆_red_*G*_gas_	∆_ox_*G*_DMSO_	∆_red_*G*_DMSO_						
**MeCN**	75-05-8	47.6	4.3	34.4	−19.8	−9.27	−0.32	35.94 [36]	355 [37]	46.4 [38]	−0.34 [39]
**DMSO**	67-68-5	−28.7	12.7	−29.6		−6.49	−0.16	46.71 [36]	464 [37]	−19.4 [38]	−1.35 [39]
**PC**	108-32-7	11.5	2.3	2.3	−14.4	−8.36	−0.31	62.93 [36]	515 [37]	50 [38]	−0.41 [39]
**SL-A0**	126-33-0	0	0	0	0	−7.89	−0.36	42.13 [36]	560 [37]	44	−0.77 [39]
**SL-A1**	397248-09-8	−2.0	−0.8	−2.6		−8.07	−0.42	47	581	62	−0.91
**SL-A2**	2413977-86-1	−2.0	−2.8			−8.21	−0.52	54	596	74	−0.44
**SL-A3**	2413977-87-2	1.1	−2.8			−8.38	−0.52	43	567	92	0.19
**SL-B0**	1192-16-1	1.5	−16.7	−2.6	−29.6	−7.92	−1.25	59	606	49	−1.42
**SL-B1a**	---	3.9	−21.6	−3.9	−36.6	−8.02	−1.22	61	601	65	−0.55
**SL-B1b**	2851432-77-2	4.3	−23.4	5.3	−35.0	−8.27	−1.60	52	597	67	−0.93
**SL-B2**	---	4.4	−29.1	6.3	−39.0	−8.45	−1.82	58	606	77	−0.28
**SL-B3**	---	10.9	−31.6	4.8	−42.1	−8.56	−1.78	50	569	97	0.78
**SL-C0**	77-79-2	1.0	−6.9	−13.8	−19.5	−8.02	−0.83	40	553	56	−0.90
**SL-C1**	444334-21-8		−23.3			−8.22	−1.63	43	565	72	−0.68
**SL-C2**	---		−27.7		−38.0	−8.41	−1.81	52	583	84	−0.03
**SL-C3**	---		−40.8		−50.7	−8.59	−2.39	42	555	100	0.59
**SL-D0**	27092-46-2	−1.9	−46.6	−9.4	−60.6	−7.66	−2.86	46	568	70	−0.63
**SL-D1**	---	−3.9	−49.4	−10.2	−62.8	−7.66	−2.89	46	545	86	0.34
**CP-A0**	120-92-3	−18.3	3.5	−19.7	−17.0	−6.83	−0.86	14.45 [36]	404 [37]	12	0.45
**CP-A1**	1755-12-0	−14.7		−15.5		−7.20	−1.63	29	457	38	0.10
**CP-A2**	2167972-33-8	−11.0	−20.9	−10.4	−40.3	−7.48	−1.87	31	456	65	0.88
**CP-B0**	930-30-3	−16.3	−15.0	−16.3	−34.2	−6.90	−1.65	33	409 [40]	−4	−0.11
**CP-B1a**	143998-28-1	−3.6	−21.6	−11.9	−38.9	−7.31	−1.94	40	484	16	0.18
**CP-B1b**	---	−11.6	−26.7	−12.2	−44.6	−7.25	−2.19	43	499	22	−0.30
**CP-B2**	---	−7.2	−34.8	−5.3	−51.1	−7.56	−2.57	46	499	44	0.40
**CP-C0**	14320-37-7	−14.8	3.4	−25.9	−18.9	−7.02	−0.96	14	399	25	0.55
**CP-C1**	175544-12-4 (R)	−10.0		−16.2		−7.37	−1.74	28	450	57	0.32
**CP-C2**	---	−5.1	−25.7	−5.3	−45.1	−7.67	−2.14	33	453	80	0.94
**CP-D0**	13177-38-3	−16.6	−48.0	−24.8	−69.1	−7.04	−3.16	22	397	47	0.86
**CP-D1**	---	−17.6	−53.8	−25.3	−72.9	−7.03	−3.40	27	403	66	1.22
**GBL-A0**	96-48-0	0.9	5.6	2.0	−14.4	−7.66	−0.23	40.96 [36]	477 [37]	22	−0.64 [39]
**GBL-A1a**	3885-31-2	5.6	2.2	6.4		−7.99	−1.11	45	504	46	−0.39
**GBL-A1b**	2343-90-0	10.2	4.3	10.0	−21.2	−8.16	−0.67	32	470	45	−0.13
**GBL-A2a**	220294-13-3	9.4	−13.7	11.2		−8.27	−1.26	44	489	64	0.33
**GBL-A2b**	1345047-11-1	17.8		17.5		−8.58	−1.00	27	452	61	0.63
**GBL-B0**	497-23-4	3.9	−17.1	−0.3	−34.2	−7.87	−1.76	56	514	21	−0.70
**GBL-B1a**	197096-95-0	11.6	−21.5	−0.2		−8.24	−1.92	55	511	40	−0.12
**GBL-B1b**	1052601-43-0	16.3	−34.7	13.5	−50.8	−8.46	−2.51	34	464	52	0.22
**GBL-B2**	24647-21-0	38.3	−44.1	22.4	−58.7	−8.92	−2.90	25	418	77	1.17
**GBL-C0**	20825-71-2	−17.9	0.9	−26.4	−19.4	−7.19	−0.94	31	437	41	0.11
**GBL-C1a**	---	−29.6		−27.6		−7.83	−2.03	30	432	76	0.60
**GBL-C1b**	1052601-43-0	−16.6		−24.8		−7.31	−1.12	22	408	61	0.88
**GBL-C2**	---	5.8	−32.9	−2.6	−52.1	−8.29	−2.44	29	404	103	1.42

## Data Availability

The data presented in this study are available in Zenodo at dx.doi.org/10.5281/zenodo.10204051 (accessed on 22 November 2023).

## Supplementary Materials

The following supporting information can be downloaded at: https://www.mdpi.com/article/10.3390/molecules28237770/s1. Table S1: ∆_tr_*G*°(H^+^) values presented in Figure 10; Table S2: Dataset used for assessment and correction of the calculated boiling points; Table S3: Dataset used for the correction of the calculated *ε* values; Figure S1: The experimental and predicted *ε_r_* values; Table S4: The estimated properties of the studied molecules; Table S5: Bond parameters of the studied solvents. Publication [84] is cited in the SI.
