# Supplementary material for: Theoretical Study on Fluorinated Derivatives of Sulfolane, Cyclopentanone, and Gamma-Butyrolactone
Source: Molecules. 2023 Nov 25;28(23):7770. doi: 10.3390/molecules28237770 (PMC10708176; doi:10.3390/molecules28237770)
Supplement: Supplementary file 1 [file molecules-28-07770-s001.zip › molecules-2609173-supplementary.pdf]

Supporting Information for

Theoretical study on fluorinated derivatives of sulfolane, cyclopentanone and gamma-butyrolactone.

by

Sofja Tshepelevitsh, Agnes Kütt, Ivo Leito

Table S 1. $\Delta_{tr}G°(H^+)$ values (kJ mol^{-1}) presented on Figure 10.

Solvent	Protonation center	$\Delta_{tr}G°$, Lit.[1]	$\Delta_{tr}G°$, COSMO-RS
Methanol	OH	10.4	-6
Ethanol	OH	11.1	-12
n-Butanol	OH	3	-14
Isopropanol	OH	9	-16
Propylene carbonate	O	50	20
N-Methyl-2-pyrrolidone	O	-25	-67
N,N-Dimethylformamide	O	-18	-54
Nitromethane	O	95	73
DMSO	O	-19.4	-53
Nitrobenzene	O	33	50
Hexamethyltriaminophosphine	P	40	-109
NH_3	N	-97	-166
Pyridine	N	-28	-107
Acetonitrile	N	46.4	30

Table S 2. Dataset used for assessment and correction of the calculated boiling points.

Compound	Exp. BP (K) [2–4]	Calc. BP (K)
Fluoroacetone	348	352
2'-Fluoroacetophenone	460	479
Phenyl trifluoromethyl ether	371	401
Methyl trifluoroacetate	317	324
Propylene carbonate	515	533
THF	338	329
Camphor (76-22-2)	481	487
Isophorone	488	519
3,4-Dihydro-2-methoxy-2H-pyran	401	398
DMSO	462	457
Dipropylsulfoxide	475	486
Dimethylsulfone	511	536
Dipropylsulfone	543	590
Sulfolane (compound SL-A0)	560	559
3-Methylsulfolane	549	608
Cyclopentanone (compound CP-A0)	404	418
Cyclopentenone (compound CP-B0)	409	476
2-Methylcyclopentanone	413	411
2-Methyl-2-cyclopentenone	433	472
Cyclohexanone	428	434
Cyclohexenone	443	460
γ-Butyrolactone (compound GBL-A0)	477	477
γ-Valerolactone	481	516
δ-Valerolactone	492	535
2-Pyrone	481	492
Succinic anhydride	537	535
Tetrafluorosuccinic anhydride	327	319
Maleic anhydride	475	464
Glutaric anhydride	563	582

Table S 3. Dataset used for correction of calculated ε values.

Nr	Set	CAS	Name	Exp. ε_r [5]	Calc. ε_r	Corrected ε_r
1	Model	392-56-3	Perfluorobenzene	2.1	1.9	2.1
2	Model	363-72-4	Pentafluorobenzene	4.4	3.3	3.8
3	Model	100-66-3	Anisole	4.5	4.2	5.0
4	Model	372-18-9	1,3-Difluorobenzene	5.2	4.1	4.9
5	Model	462-06-6	Fluorobenzene	5.6	4.3	5.3
6	Model	109-99-9	THF	7.5	5.9	7.5
7	Model	110-01-0	Tetrahydrothiophene	8.6	7.7	10.0
8	Model	98-08-8	Trifluoromethylbenzene	9.4	8.0	10.5
9	Model	95-50-1	1,2-Dichlorobenzene	10.4	8.5	11.2
10	Model	367-11-3	1,2-Difluorobenzene	14.3	7.9	10.2
11	Model	120-92-3	Cyclopentanone (CP-A0)	14.5	12.7	17.5
12	Model	108-94-1	Cyclohexanone	16.0	13.0	18.1
13	Model	98-86-2	Acetophenone	18.2	11.5	15.7
14	Model	872-93-5	3-Methylsulfolane	29.4	28.3	43.1
15	Model	542-28-9	γ-Valerolactone	36.1	25.0	37.5
16	Model	96-48-0	γ-Butyrolactone (GBL-A0)	41.0	27.3	41.3
17	Model	126-33-0	Sulfolane (SL-A0)	42.1	31.2	48.0
18	Model	1600-44-8	Tetramethylene sulfoxide	42.8	24.6	36.8
19	Model	108-32-7	Propylene carbonate	62.9	36.5	57.2
20	Model	96-49-1	Ethylene carbonate	89.8	39.2	62.0
21	Validation	105-58-8	Diethyl carbonate	2.9	5.1	6.3
22	Validation	616-38-6	Dimethyl carbonate	3.2	6.2	7.9
23	Validation	141-78-6	Ethyl acetate	6.0	6.0	7.6
24	Validation	554-12-1	Methyl propionate	6.2	6.1	7.7
25	Validation	110-74-7	Propyl formate	7.7	8.0	10.4
26	Validation	515-84-4	Ethyl trichloroacetate	9.0	8.6	11.4
27	Validation	918-00-3	1,1,1-Trichloroacetone	10.4	7.5	9.7
28	Validation	431-47-0	Methyl trifluoroacetate	11.5	9.9	13.2
29	Validation	96-22-0	3-Pentanone	17.5	9.7	12.9
30	Validation	78-93-3	2-Butanone	18.9	11.4	15.5
31	Validation	123-54-6	Acetylacetone	25.7	12.4	17.1
32	Validation	872-50-4	NMP	32.6	25.4	38.1
33	Validation	75-05-8	Acetonitrile	35.9	25.3	38.1
34	Validation	98-95-3	Nitrobenzene	36.1	22.4	33.1
35	Validation	75-52-5	Nitromethane	36.2	18.0	25.9
36	Validation	372-48-5	2-Fluoropyridine	37.3	14.5	20.4
37	Validation	127-19-5	DMAC	38.3	23.9	35.6
38	Validation	67-68-5	DMSO	46.7	24.6	36.8
39	Validation	77-78-1	Dimethyl sulfate	50.3	20.0	29.1
40	Validation	1513-65-1	2,6-Difluoropyridine	107.8	16.7	23.9

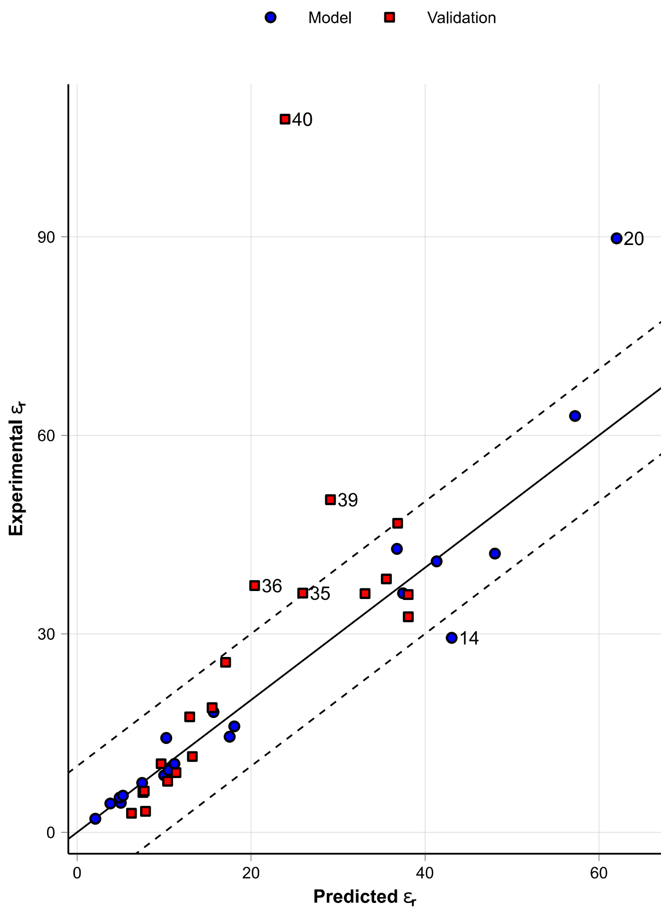

Figure S 1. Experimental and predicted ε_r values. Solid line is the diagonal of the plot. Dashed lines mark -10 and +10 deviations. The compounds with absolute errors over 10 are labeled according to their numbers in Table S 3.

Table S 4. Estimated properties of the studied molecules. Where available, experimental values are given in parentheses. $\Delta_{ox}G$ and $\Delta_{red}G$ values are relative to sulfolane (SL-A0). Solubility, ε_r, and $\Delta_{tr}G°(H^+)$ values are computed with the assumption that the solvent is liquid at room temperature.

Compound	CAS	Core structure	Subst.	Relative red./ox. energies (kcal mol^{-1})				E_{HOMO} (eV)	E_{LUMO} (eV)	ε_r	BP (K)	$\Delta_{tr}G°(H^+)$ (kJ mol^{-1})	log$P_{o/w}$	pK_{aH} in MeCN	GB (kcal mol^{-1})	Solub. of water in solvent (x)	Solub. in water (x)
				$\Delta_{ox}G_{gas}$	$\Delta_{red}G_{gas}$	$\Delta_{ox}G_{DMSO}$	$\Delta_{red}G_{DMSO}$										
**SL-A0**	126-33-0	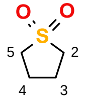	—	0	0	0	0	-7.89	-0.36	48 (42.13 [5])	570 (560 [3])	44	-1.31 (-0.77 [6])	-7	188.1	misc.	misc.
**SL-A1**	397248-09-8		**2-F**	-2.0	-0.8	-2.6		-8.07	-0.42	47	580	62	-0.91	-10	183.2	0.287	0.051
**SL-A2**	2413977-86-1		**2,2-F$_2$**	-2.0	-2.8			-8.21	-0.52	54	595	74	-0.44	-12	179.5	0.111	0.012
**SL-A3**	2413977-87-2		**2,2,5-F$_3$**	1.1	-2.8			-8.38	-0.52	43	566	92	0.19	-15	173.2	0.048	0.004
**SL-B0**	1192-16-1	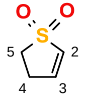	—	1.5	-16.7	-2.6	-29.6	-7.92	-1.25	59	605	49	-1.42	-7	189.1	misc.	misc.
**SL-B1a**	---		**2-F**	3.9	-21.6	-3.9	-36.6	-8.02	-1.22	61	600	65	-0.55	-11	182.9	0.160	0.022
**SL-B1b**	2851432-77-2		**5-F**	4.3	-23.4	5.3	-35.0	-8.27	-1.60	52	596	67	-0.93	-11	182.5	0.212	0.035
**SL-B2**	---		**5,5-F$_2$**	4.4	-29.1	6.3	-39.0	-8.45	-1.82	58	604	77	-0.28	-13	178.7	0.089	0.010
**SL-B3**	---		**2,5,5-F$_3$**	10.9	-31.6	4.8	-42.1	-8.56	-1.78	50	567	97	0.78	-16	172.1	0.031	0.002
**SL-C0**	77-79-2	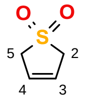	—	1.0	-6.9	-13.8	-19.5	-8.02	-0.83	40	552	56	-0.90	-9	184.8	0.488	0.133
**SL-C1**	444334-21-8		**2-F**		-23.3			-8.22	-1.63	43	564	72	-0.68	-12	180.4	0.175	0.026
**SL-C2**	---		**2,2-F$_2$**		-27.7		-38.0	-8.41	-1.81	52	582	84	-0.03	-14	176.3	0.073	0.007
**SL-C3**	---		**2,2,5-F$_3$**		-40.8		-50.7	-8.59	-2.39	42	554	100	0.59	-17	170.3	0.036	0.003
**SL-D0**	27092-46-2	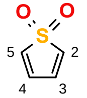	—	-1.9	-46.6	-9.4	-60.6	-7.66	-2.86	46	566	70	-0.63	-12	182.1	0.197	0.040
**SL-D1**	---		**2-F**	-3.9	-49.4	-10.2	-62.8	-7.66	-2.89	46	544	86	0.34	-14	176.3	0.054	0.007
**CP-A0**	120-92-3	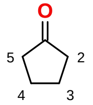	—	-18.3	3.5	-19.7	-17.0	-6.83	-0.86	18 (14.45 [5])	408 (404 [3])	12	0.45	-2	190.0 (189.8 [2])	0.485	0.087
**CP-A1**	1755-12-0		**2-F**	-14.7		-15.5		-7.20	-1.63	29	456	38	0.10	-5	185.2	0.207	0.035
**CP-A2**	2167972-33-8		**2,2-F$_2$**	-11.0	-20.9	-10.4	-40.3	-7.48	-1.87	31	455	65	0.88	-10	177.2	0.032	0.003
**CP-B0**	930-30-3	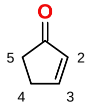	—	-16.3	-15.0	-16.3	-34.2	-6.90	-1.65	33	458 (409 [2])	-4	-0.11	2	196.5	misc.	misc.
**CP-B1a**	143998-28-1		**2-F**	-3.6	-21.6	-11.9	-38.9	-7.31	-1.94	40	483	16	0.18	-1	191.1	0.309	0.037
**CP-B1b**	---		**5-F**	-11.6	-26.7	-12.2	-44.6	-7.25	-2.19	43	498	22	-0.30	-2	189.9	0.387	0.080
**CP-B2**	---		**5,5-F$_2$**	-7.2	-34.8	-5.3	-51.1	-7.56	-2.57	46	498	44	0.40	-6	183.3	0.067	0.007
**CP-C0**	14320-37-7	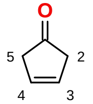	—	-14.8	3.4	-25.9	-18.9	-7.02	-0.96	14	398	25	0.55	-4	185.8	0.268	0.033
**CP-C1**	175544-12-4 (R)		**2-F**	-10.0		-16.2		-7.37	-1.74	28	449	57	0.32	-9	179.8	0.082	0.013
**CP-C2**	---		**2,2-F$_2$**	-5.1	-25.7	-5.3	-45.1	-7.67	-2.14	33	452	80	0.94	-12	173.3	0.021	0.002
**CP-D0**	13177-38-3	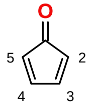	—	-16.6	-48.0	-24.8	-69.1	-7.04	-3.16	22	396	47	0.86	-7	182.9	0.059	0.009
**CP-D1**	---		**2-F**	-17.6	-53.8	-25.3	-72.9	-7.03	-3.40	27	403	66	1.22	-10	177.4	0.023	0.003

Compound	CAS	Core structure	Subst.	Relative red./ox. energies (kcal mol^{-1})				E_{HOMO} (eV)	E_{LUMO} (eV)	ε_r	BP (K)	$\Delta_{tr}G°(H^+)$ (kJ mol^{-1})	$\log P_{o/w}$	pK_{aH} in MeCN	GB (kcal mol^{-1})	Solub. of water in solvent (x)	Solub. in water (x)
				$\Delta_{ox}G_{gas}$	$\Delta_{red}G_{gas}$	$\Delta_{ox}G_{DMSO}$	$\Delta_{red}G_{DMSO}$										
**GBL-A0**	96-48-0	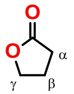	—	0.9	5.6	2.0	-14.4	-7.66	-0.23	41 (40.96 [5])	481 (477 [3])	22	-0.47 (-0.64 [6])	-3	193.5 (193.1 [2])	misc.	misc.
**GBL-A1a**	3885-31-2		**α-F**	5.6	2.2	6.4		-7.99	-1.11	45	503	46	-0.39	-7	185.5	0.162	0.032
**GBL-A1b**	2343-90-0		**γ-F**	10.2	4.3	10.0	-21.2	-8.16	-0.67	32	469	45	-0.13	-6	185.5	0.120	0.025
**GBL-A2a**	220294-13-3		**α,α-F_2**	9.4	-13.7	11.2		-8.27	-1.26	44	488	64	0.33	-10	179.6	0.045	0.006
**GBL-A2b**	1345047-11-1		**γ,γ-F_2**	17.8		17.5		-8.58	-1.00	27	451	61	0.63	-9	179.9	0.034	0.005
**GBL-B0**	497-23-4	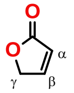	—	3.9	-17.1	-0.3	-34.2	-7.87	-1.76	56	513	21	-0.70	-2	193.7	misc.	misc.
**GBL-B1a**	197096-95-0		**α-F**	11.6	-21.5	-0.2		-8.24	-1.92	55	510	40	-0.12	-6	186.1	0.168	0.032
**GBL-B1b**	1052601-43-0		**γ-F**	16.3	-34.7	13.5	-50.8	-8.46	-2.51	34	463	52	0.22	-8	182.9	0.073	0.015
**GBL-B2**	24647-21-0		**γ,γ-F_2**	38.3	-44.1	22.4	-58.7	-8.92	-2.90	25	417	77	1.17	-11	175.5	0.020	0.003
**GBL-C0**	20825-71-2	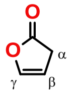	—	-17.9	0.9	-26.4	-19.4	-7.19	-0.94	31	436	41	0.11	-6	186.5	0.138	0.033
**GBL-C1a**	---		**α-F**	-29.6		-27.6		-7.83	-2.03	30	431	76	0.60	-12	176.0	0.032	0.007
**GBL-C1b**	1052601-43-0		**γ-F**	-16.6		-24.8		-7.31	-1.12	22	407	61	0.88	-9	178.7	0.032	0.005
**GBL-C2**	---		**α,α-F_2**	5.8	-32.9	-2.6	-52.1	-8.29	-2.44	29	403	103	1.42	-16	169.1	0.012	0.002

Table S 5. Bond parameters of the studied solvents. Structures were optimized at B3LYP/6-311+G level of theory. Bond numbering is shown on Figures 2-4 in the main text.**

Compound	Length (Å)						Wiberg bond index						Intrinsic bond strength index					
	1-2	2-3	3-4	4-5	5-1	1-6	1-2	2-3	3-4	4-5	5-1	1-6	1-2	2-3	3-4	4-5	5-1	1-6
SL-A0	1.843	1.530	1.544	1.530	1.843	1.469	0.899	1.103	1.072	1.103	0.899	1.865	0.346	0.573	0.548	0.573	0.346	0.845
SL-A1	1.875	1.514	1.546	1.535	1.847	1.466	0.817	1.091	1.061	1.096	0.894	1.873	0.318	0.602	0.544	0.565	0.343	0.853
SL-A2	1.927	1.512	1.542	1.533	1.837	1.463	0.718	1.046	1.058	1.095	0.900	1.885	0.277	0.602	0.549	0.569	0.352	0.862
SL-A3	1.931	1.514	1.543	1.519	1.875	1.460	0.713	1.044	1.048	1.082	0.812	1.894	0.274	0.600	0.546	0.594	0.318	0.869
SL-B0	1.790	1.326	1.513	1.536	1.843	1.467	0.928	1.954	1.085	1.088	0.895	1.863	0.404	1.110	0.596	0.561	0.346	0.849
SL-B1a	1.814	1.322	1.512	1.541	1.833	1.464	0.838	1.861	1.075	1.080	0.900	1.875	0.377	1.117	0.599	0.552	0.357	0.858
SL-B1b	1.791	1.326	1.515	1.524	1.878	1.464	0.922	1.952	1.074	1.072	0.812	1.874	0.404	1.110	0.592	0.581	0.316	0.857
SL-B2	1.787	1.327	1.512	1.521	1.920	1.461	0.924	1.945	1.072	1.028	0.721	1.883	0.408	1.103	0.597	0.585	0.283	0.865
SL-B3	1.812	1.322	1.512	1.524	1.916	1.457	0.835	1.853	1.061	1.024	0.720	1.894	0.380	1.113	0.596	0.581	0.286	0.873
SL-C0	1.846	1.498	1.334	1.498	1.846	1.467	0.893	1.119	1.931	1.119	0.893	1.874	0.342	0.629	1.080	0.629	0.342	0.847
SL-C1	1.878	1.491	1.335	1.499	1.840	1.464	0.813	1.096	1.906	1.115	0.891	1.883	0.315	0.642	1.074	0.627	0.348	0.858
SL-C2	1.922	1.487	1.332	1.499	1.840	1.461	0.723	1.056	1.894	1.113	0.889	1.894	0.281	0.648	1.079	0.624	0.348	0.866
SL-C3	1.919	1.490	1.332	1.495	1.881	1.457	0.721	1.048	1.882	1.083	0.804	1.904	0.283	0.642	1.077	0.632	0.312	0.874
SL-D0	1.807	1.332	1.485	1.332	1.807	1.465	0.896	1.924	1.118	1.924	0.896	1.875	0.386	1.089	0.652	1.089	0.386	0.852
SL-D1	1.838	1.327	1.481	1.334	1.798	1.461	0.800	1.827	1.114	1.907	0.905	1.887	0.352	1.095	0.660	1.081	0.395	0.861
CP-A0	1.531	1.537	1.547	1.537	1.531	1.207	1.034	1.076	1.071	1.076	1.034	2.411	0.566	0.561	0.545	0.561	0.566	1.312
CP-A1	1.543	1.524	1.546	1.542	1.527	1.202	0.974	1.052	1.061	1.068	1.033	2.428	0.546	0.580	0.547	0.553	0.573	1.330
CP-A2	1.562	1.514	1.546	1.543	1.522	1.198	0.902	1.033	1.055	1.064	1.035	2.440	0.515	0.598	0.545	0.550	0.580	1.343
CP-B0	1.481	1.339	1.510	1.541	1.536	1.212	1.109	1.856	1.095	1.063	1.021	2.344	0.660	1.062	0.607	0.552	0.559	1.291
CP-B1a	1.483	1.334	1.508	1.547	1.529	1.208	1.043	1.761	1.087	1.054	1.022	2.353	0.653	1.070	0.610	0.543	0.571	1.307
CP-B1b	1.477	1.340	1.509	1.533	1.551	1.207	1.111	1.841	1.087	1.038	0.961	2.362	0.670	1.058	0.606	0.564	0.535	1.307
CP-B2	1.472	1.341	1.509	1.529	1.566	1.204	1.113	1.829	1.082	1.007	0.897	2.372	0.678	1.053	0.606	0.572	0.511	1.320
CP-C0	1.536	1.506	1.336	1.506	1.536	1.205	1.024	1.092	1.936	1.092	1.024	2.425	0.556	0.615	1.082	0.615	0.556	1.319
CP-C1	1.553	1.496	1.336	1.506	1.531	1.200	0.958	1.077	1.905	1.089	1.021	2.447	0.529	0.633	1.076	0.614	0.563	1.336
CP-C2	1.564	1.490	1.335	1.507	1.526	1.197	0.899	1.045	1.885	1.083	1.020	2.455	0.513	0.642	1.076	0.610	0.571	1.349

Compound	Length (Å)						Wiberg bond index						Intrinsic bond strength index					
	1-2	2-3	3-4	4-5	5-1	1-6	1-2	2-3	3-4	4-5	5-1	1-6	1-2	2-3	3-4	4-5	5-1	1-6
CP-D0	1.511	1.338	1.503	1.338	1.511	1.209	1.032	1.891	1.085	1.891	1.032	2.376	0.606	1.072	0.625	1.072	0.606	1.292
CP-D1	1.526	1.331	1.502	1.341	1.498	1.204	0.951	1.808	1.075	1.865	1.049	2.391	0.575	1.085	0.627	1.061	0.628	1.310
GBL-A0	1.525	1.531	1.535	1.445	1.364	1.197	1.003	1.073	1.071	1.197	1.415	2.377	0.574	0.568	0.561	0.591	0.759	1.349
GBL-A1a	1.542	1.515	1.536	1.449	1.357	1.195	0.935	1.066	1.062	1.186	1.431	2.384	0.545	0.596	0.560	0.582	0.777	1.357
GBL-A1b	1.521	1.531	1.527	1.404	1.386	1.191	1.005	1.064	1.043	1.276	1.329	2.415	0.579	0.568	0.574	0.675	0.703	1.373
GBL-A2a	1.553	1.512	1.534	1.454	1.352	1.191	0.875	1.026	1.056	1.178	1.438	2.395	0.528	0.599	0.562	0.572	0.788	1.374
GBL-A2b	1.520	1.531	1.526	1.390	1.392	1.188	1.006	1.060	0.996	1.269	1.301	2.430	0.581	0.567	0.575	0.707	0.686	1.385
GBL-B0	1.484	1.332	1.499	1.430	1.379	1.198	1.058	1.879	1.089	1.220	1.360	2.346	0.652	1.084	0.622	0.621	0.724	1.342
GBL-B1a	1.487	1.327	1.499	1.440	1.369	1.195	0.986	1.783	1.080	1.201	1.380	2.356	0.641	1.091	0.624	0.602	0.748	1.357
GBL-B1b	1.488	1.328	1.507	1.405	1.389	1.192	1.037	1.888	1.034	1.260	1.311	2.388	0.642	1.095	0.608	0.675	0.699	1.363
GBL-B2	1.489	1.327	1.510	1.386	1.399	1.188	1.029	1.883	0.981	1.272	1.270	2.414	0.639	1.098	0.604	0.719	0.674	1.379
GBL-C0	1.526	1.501	1.330	1.389	1.389	1.192	1.003	1.084	1.898	1.300	1.332	2.407	0.571	0.621	1.095	0.703	0.697	1.368
GBL-C1a	1.548	1.492	1.330	1.388	1.386	1.187	0.930	1.071	1.870	1.300	1.328	2.441	0.536	0.640	1.089	0.704	0.703	1.387
GBL-C1b	1.525	1.505	1.325	1.368	1.405	1.188	1.005	1.073	1.785	1.299	1.279	2.435	0.574	0.616	1.102	0.753	0.659	1.386
GBL-C2	1.563	1.487	1.330	1.390	1.381	1.183	0.864	1.040	1.852	1.294	1.332	2.453	0.513	0.648	1.089	0.699	0.715	1.400

References

1. Marcus, Y.; Kamlet, M.J.; Taft, R.W. Linear Solvation Energy Relationships: Standard Molar Gibbs Free Energies and Enthalpies of Transfer of Ions from Water into Nonaqueous Solvents. *J. Phys. Chem.* **1988**, *92*, 3613–3622, doi:10.1021/j100323a057.
2. *NIST Chemistry WebBook, NIST Standard Reference Database Number 69*; Linstrom, P.J., Mallard, W.G., Eds.; National Institute of Standards and Technology: Gaithersburg MD, 20899, https://doi.org/10.18434/T4D303, (retrieved November 24, 2023).
3. Yaws, C.L. *Thermophysical Properties of Chemicals and Hydrocarbons*; William Andrew: Norwich, NY, 2008; ISBN 978-0-8155-1596-8.
4. Reinisch, J.; Klamt, A. Predicting Flash Points of Pure Compounds and Mixtures with COSMO-RS. *Ind. Eng. Chem. Res.* **2015**, *54*, 12974–12980, doi:10.1021/acs.iecr.5b03083.
5. Abboud, J.-L.M.; Notari, R. Critical Compilation of Scales of Solvent Parameters. Part I. Pure, Non-Hydrogen Bond Donor Solvents. *Pure Appl. Chem.* **1999**, *71*, 645–718, doi:10.1351/pac199971040645.
6. Hansch, C.; Leo, A.; Hoekman, D. *Exploring QSAR: Hydrophobic, Electronic, and Steric Constants*; ACS professional reference book; American Chemical Society: Washington, DC, 1995; ISBN 978-0-8412-2987-7.
